# In Nature's School

**Published:** 1889-07-06

**Authors:** 


### The Doctor's Pulpit.

### IN NATURE'S SCHOOL.

### Room to Breathe.

Never was there greater wealth of vegetation in "Merrie England" than at the present time. It needs the pen of a poet to adequately describe the fields and the hedgerows; the oak, the ash, and the beech; the buttercups and daisies now passing away; the scarlet pimpernels and the forget-me-nots; the cranesbills and the ragged robins; the meadow-sweets, and the red-flushing poppies in the corn. The present writer is painfully conscious of the lack of descriptive powers; but even a Ruskin might well despair of painting a word picture which should worthily present to the mind the glorious abundance of leaf and flower which bursts on the sight on every hand. In spite of all the thick studding of field and copse with tree and shrub, grass and flower, there seems to be no want of space; no elbowing; no starving of diminutive shrubs by overgrown trees; no killing of the smaller and less robust flowers by their larger and more spreading fellow tenants of the meadow and the green banks. In the rich glow of the generous summer all alike have shared; each has had its needed measure of sunshine, rain, and dew; and each has shown its native energy and life by putting forth its broadest and greenest leaves and its brightest and rarest flowers. So expands a mighty state in the sunshine of national prosperity; so prospers a family in the vigorous well-being of its head; so develop all good qualities in man, woman, or child when body and mind are in the joyous robustness of wholesome and perfect health.

"Room to breathe" is one of the most urgent necessities of the times. Capable men and women of all classes and conditions are becoming distressingly numerous and crowded. Like a vast army of flies in a small hopfield, there is hardly leaf-space for them to rest upon, much less enough of the succulent green to supply the daily hunger of all. The hopgrower makes short work of the flies with his black soap and his spray-distributor; but in a rational community the forces of Nature are kept well in check, and men multiply faster than the means of subsistence, or at any rate too quickly for the ordinary methods of food distribution. How is it that Nature in a summer like the present manages so much better with her excess of vegetable life than men do with the excess of population? We crowd upon one another; we fight for standing room and for the great and small rewards that are attainable; we trample each other in the dust and mire without the smallest hesitation or compunction. The least competitive and most peace-loving of men and women find themselves stripped and fighting in the world's arena before they have had time to look about them. It cannot be said that all this makes for either happiness or well-being. Even the successful find themselves so battered and bruised on all sides by their less fortunate rivals that they are often tempted to throw up the struggle, and to declare that no reward the world has to offer is worth the labour and toil and strife and hatred which seem to be inevitable accompaniments of the winning of it.

Men appear to have almost as little control over their destinies as have the trees and the beasts of the field. There is a compulsion of increase and a compulsion of struggle which none of us are able to resist. A deep-thinking man like Malthus or Mill fondly imagines he has discovered and made plain to others certain regulative principles which will lessen the fierceness of the strife, and give both the strong and the weak juster and more satisfying rewards for their labours. But what practical result on a large scale ever comes from the regulative principles of either Malthus or Mill? The secret of the more uniform prosperity of the vegetable world appears to be that there is no fighting among vegetables. The smaller trees and flowers do not envy and hate the larger as small men are apt to do. If they were accustomed to think and express their thoughts, one could imagine the scarlet pimpernel saying to the oxeye—"You are larger than I am, and perhaps you may please some persons better and serve a different purpose in life; but though I am small many people think me pretty, and children are often delighted when they see a little scarlet creature peeping out in places where they had often looked carelessly before without seeing a flower at all. Besides, although I am not so big as an oxeye, I am quite as big and as pretty as any of my fellow pimpernels; and at any rate the soil supplies me with all the solid food I really need, the rain and the dew visit me whenever they visit the other plants and flowers, and all day long and all night through the sweet air plays about me just the same as it does about the ashes, and the oaks, and the pines. I am quite well, and happy to be a pimpernel; I hope you are quite well, and content to be an oxeye." That is the plain sense of the business. The pimpernel does not, because it finds itself small, begin to rail at and attack bigger plants than itself as human creatures often do. If it did we might find it some day engaged in mortal conflict with the oak. What it does do is to lay firmly hold of the common sources of supply by means of its own organs, and to gather in the nutriment it wants from day to day by its own efforts. Its roots select what they need from the soil, its leaves and flowers spread themselves to catch the dew and the rain, the air, and the rays of the sun. Thus, living side by side, the small pimpernel, the larger oxeye, and the giant oak are alike prosperous, contented, and altogether brotherly with one another.

The writer is not forgetting that in the animal world a somewhat different order of things prevails; and that, too, will receive consideration in due time. His present object is to show how much of well-being may result from every person minding his own business; doing his best in his own sphere, and putting out of his mind and heart the envy and jealousy of his fellows, which are so certain to poison the happiest temper and ruin the best disposition. After all, a man must be what he can be. If he be born an ox he will certainly develop into an ox if he lives, whether he is born in a cottager's cowhouse or a prince's lordly cattle lair. But if he have been born a frog and not an ox, a frog he will remain through life, though he should distend his flanks to the capacity of a balloon and his head to the dimensions of the car that is suspended therefrom. But what then? If a frog is not an ox, and never can be, it may yet be a very fine, fat, and prosperous frog, for all that. The frogs of the present day might even turn round on the bullocks and claim to be nobler animals than they, on the ground that whilst the bullock associates with the cowboy and the butcher, the frog finds his human companions among scientific men ; and though, like the bullock, he needs must die, yet he dies in the physiological laboratory, in the great cause of science, whilst the bullock sheds his blood in the coarsest way in the butcher's shop, and is converted into sirloins and tripe.

The lesson of laying hold of the common sources of supply, each one for himself, like the plants, and of ceasing to lay hold of the throats of one's bigger neighbours, is well worth understanding and thinking about. Common men, it is often thought, have all the green gooseberries of life, whilst the wealthy and the cultured have all the peaches and the apricots. But it is not really so. People are so terribly stupid. In passing through the world's fair nine out of ten will not know the difference between a real peach and a painted one, and when they see so many painted gauds on the stalls of the rich they cry out that the prosperous have got all the best fruit. What are the real peaches of life ? Are they not good health, sound sleep, appetite and digestion, an honest conscience, a contented mind, and the like ? Will anyone contend that the throne of England is always a peach to the Queen ; that the woolsack is never a thorny seat to the Lord Chancellor ; that a Rothschild does not sometimes find his wealth green gooseberries ? Even the parish beadle, the greatest of all great beings, may often prove in his own sorrowful experience how "uneasy is the head that wears a crown." If we persist in all rushing together into one common *melee*, the world will undoubtedly seem small and the breathing room distressingly limited. But if, like the grass, the flowers, and the trees, we each resolve to do his utmost share in laying hold of the common sources of wealth and prosperity, in subduing Nature for the good, not only of the individual but of the community, it is certain that the happiness of each and the prosperity of all will be very largely secured.

The problems of life are not to be solved to any great extent by men acting collectively, but by men thinking and acting individually. Individual thought and action does not imply separation, isolation and selfishness. It implies a clear understanding that each man must ordinarily depend upon his own efforts, though in certain spheres the intelligent and concerted efforts of all will best meet the difficulty. Without thought there can be no individuality, and without individuality there can be no concerted purpose and action. The individual must be dealt with first of all, and he must deal with himself above all, or permanent common prosperity is impossible. Perhaps no one sees these things so clearly as the thinking medical man. He sees, day by day, that health is peculiar to the individual, and that disease, too, is peculiar. If the husband have a diseased heart, the wife can neither give him a sound one nor endure the pain for him. If the wife be suffering from cancer, the husband cannot, by the most devoted affection, substitute his robust vigour for her pining health and failing strength. If the child be wasting away in consumption, the mother, though she should lay her life down a hundred times a day, cannot breathe new lungs into the shrinking chest or new life into the fainting heart. Individuality is the note of every human being—an individual body, an individual soul, individual responsibility, individual work. The world supplies all, as it supplies the trees and the plants, with an arena, breathing room, and certain common sources of life and health. If a man would have the peaches and not the green gooseberries of life, there is one way of obtaining them in perfection, and only one. He must recognise that he is in Nature's school, that her lessons are there to be learned and practised, and that according as he learns and practises these will his reward or punishment be. The well-disposed man, who means to do the best he can, always with due regard to his neighbour's claims and rights, seldom finds himself much cramped for breathing room. The man of the opposite disposition, who wants all the world to himself, is always in collisions, always in strife, often in disappointment, and, practically, never at peace.

